# Immediate and delayed autologous abdominal microvascular flap breast reconstruction in patients receiving adjuvant, neoadjuvant or no radiotherapy: a meta‐analysis of clinical and quality‐of‐life outcomes

**DOI:** 10.1002/bjs5.50245

**Published:** 2019-12-29

**Authors:** A. Khajuria, W. N. Charles, M. Prokopenko, A. Beswick, A. L. Pusic, A. Mosahebi, D. J. Dodwell, Z. E. Winters

**Affiliations:** ^1^ Kellogg College, Nuffield Department of Surgery University of Oxford Oxford UK; ^2^ Nuffield Department of Population Health University of Oxford Oxford UK; ^3^ Department of Surgery and Cancer Imperial College London London UK; ^4^ Department of Plastic Surgery Royal Free Hospital London UK; ^5^ Surgical Intervention Trials Unit, Division of Surgery and Interventional Science University College London London UK; ^6^ School of Clinical Sciences University of Bristol Bristol UK; ^7^ Patient‐Reported Outcomes, Value and Experience Centre, Brigham and Women's Hospital Harvard Medical School Boston Massachusetts USA

## Abstract

**Background:**

Effects of postmastectomy radiotherapy (PMRT) on autologous breast reconstruction (BRR) are controversial regarding surgical complications, cosmetic appearance and quality of life (QOL). This systematic review evaluated these outcomes after abdominal free flap reconstruction in patients undergoing postoperative adjuvant radiotherapy (PMRT), preoperative radiotherapy (neoadjuvant radiotherapy) and no radiotherapy, aiming to establish evidence‐based optimal timings for radiotherapy and BRR to guide contemporary management.

**Methods:**

The study was registered on PROSPERO (CRD42017077945). Embase, MEDLINE, Google Scholar, CENTRAL, Science Citation Index and ClinicalTrials.gov were searched (January 2000 to August 2018). Study quality and risk of bias were assessed using GRADE and Cochrane's ROBINS‐I respectively.

**Results:**

Some 12 studies were identified, involving 1756 patients (350 PMRT, 683 no radiotherapy and 723 neoadjuvant radiotherapy), with a mean follow‐up of 27·1 (range 12·0–54·0) months for those having PMRT, 16·8 (1·0–50·3) months for neoadjuvant radiotherapy, and 18·3 (1·0–48·7) months for no radiotherapy. Three prospective and nine retrospective cohorts were included. There were no randomized studies. Five comparative radiotherapy studies evaluated PMRT and four assessed neoadjuvant radiotherapy. Studies were of low quality, with moderate to serious risk of bias. Severe complications were similar between the groups: PMRT *versus* no radiotherapy (92 *versus* 141 patients respectively; odds ratio (OR) 2·35, 95 per cent c.i. 0·63 to 8·81, *P* = 0·200); neoadjuvant radiotherapy *versus* no radiotherapy (180 *versus* 392 patients; OR 1·24, 0·76 to 2·04, *P* = 0·390); and combined PMRT plus neoadjuvant radiotherapy *versus* no radiotherapy (272 *versus* 453 patients; OR 1·38, 0·83 to 2·32, *P* = 0·220). QOL and cosmetic studies used inconsistent methodologies.

**Conclusion:**

Evidence is conflicting and study quality was poor, limiting recommendations for the timing of autologous BRR and radiotherapy. The impact of PMRT and neoadjuvant radiotherapy appeared to be similar.

## Graphical abstract

A PROSPERO‐registered, PRISMA‐compliant meta‐analysis was conducted on clinical and patient‐reported outcomes of immediate *versus* delayed abdominal‐based free‐flap breast reconstruction, in the context of radiotherapy. Current evidence is conflicting, with no consistent recommendations for autologous breast reconstruction and radiotherapy. High‐quality evidence is mandatory for national guidelines and to optimize informed consent.

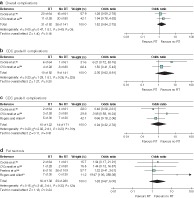

Better quality evidence still needed.

## Abstract (es)

**Antecedentes:**

En pacientes sometidas a una reconstrucción mamaria (*breast reconstruction*, BRR) con tejido autólogo se discuten los efectos de la radioterapia post‐mastectomía (*post‐mastectomy radiotherapy*, PMRT) en las complicaciones quirúrgicas, el resultado estético y la calidad de vida (*quality of life*, QOL). Esta revisión sistemática evaluó dichos resultados tras una reconstrucción mamaria con un colgajo libre abdominal en pacientes tratadas con PMRT, radioterapia preoperatoria (Neo RT) y sin radioterapia (RT), a fin de establecer los momentos óptimos de la RT y BRR basados en la evidencia, como guía del tratamiento actual.

**Métodos:**

El estudio se registró en la base de datos PROSPERO (CRD42017077945). Se realizaron búsquedas en Embase, MEDLINE, Google Scholar, CENTRAL, Science Citation Index y Clinicaltrials.gov (enero de 2000‐agosto de 2018). La calidad de los estudios y el riesgo de sesgo se evaluaron mediante las herramientas GRADE y ROBINS‐I de la Cochrane, respectivamente.

**Resultados:**

Se identificaron 12 estudios que incluían 1.756 pacientes (350 PMRT, 683 sin RT y 723 Neo RT), con una mediana de seguimiento de 27,1 meses (rango 12,0‐54,0) para PMRT, 16,8 meses (1,0‐50,3) para Neo RT y 18,3 meses (1,0‐48,7) para sin RT. Se incluyeron tres cohortes prospectivas y nueve retrospectivas. No hubo estudios aleatorizados. Los estudios comparativos de RT evaluaron la PMRT (*n* = 5) y la Neo RT (*n* = 4). Todos los estudios fueron de baja calidad, con riesgos de sesgo de moderados a graves. Las complicaciones graves fueron similares entre los grupos: PMRT (n = 92) *versus* sin RT (n = 141), razón de oportunidades (*odds ratio*, OR) 2,35, i.c. del 95% 0,63‐8,81), *P* = 0,200; Neo RT (*n* = 180) *versus* no RT (*n* = 392) (OR 1,24, i.c. del 95% 0,76‐2,04), *P* = 0,390; o RT combinada (PMRT y neoadyuvante) (*n* = 272) *versus* no RT (*n* = 453) (OR 1,38, i.c. del 95% 0,83‐2,32), *P* = 0,220. Los estudios de calidad de vida y de resultados estéticos utilizaron metodologías poco consistentes.

**Conclusión:**

La evidencia es contradictoria y la calidad de los estudios muy pobre, hechos que limitan las posibles recomendaciones para el momento de la BRR con tejido autólogo y la RT. El impacto de la PMRT o la Neo RT parecen ser similares.

## Introduction

Breast cancer is the commonest malignancy and leading cause of cancer‐related mortality in women^1^, ^2^. Breast‐conserving surgery (BCS) with radiotherapy or mastectomy are recommended treatments, with comparable oncological outcomes^3^, ^4^. Autologous abdominal‐based free flap and implant‐based procedures are the approaches used most frequently in immediate breast reconstruction (BRR)^5^. Autologous BRR has the inherent advantage of using the patient's own tissues, taken from a different part of the body where there is excess fat and skin, to restore breast volume and appearance after mastectomy. Various donor sites can be used, most commonly the abdomen^6^.

Adjuvant locoregional postmastectomy radiotherapy (PMRT) of the chest wall, and potentially of the regional lymph nodes, has been indicated historically for locally advanced disease^7^, ^8^. These indications increased following the Early Breast Cancer Trialists' Collaborative Group^9^ meta‐analyses, which showed significantly improved disease‐free and overall survival after PMRT and regional node irradiation in women at intermediate risk (tumour size 50 mm or less and 1–3 positive lymph nodes)^10^. Newly proposed US guidelines^11^ emphasize the need to consider the lower recurrence rates associated with contemporary practice and the benefits of systemic therapy^12^. Current recommendations for PMRT in the intermediate‐risk group remain controversial, pending the results of the SUPREMO (Selective Use of Postoperative Radiotherapy aftEr MastectOmy) trial, evaluating chest wall and/or axillary radiotherapy^13^, ^14^.

Adjuvant radiotherapy (PMRT) may have deleterious effects on breast cosmetic outcomes, quality of life (QOL) and surgical complications after immediate BRR^15^. Previous studies evaluating the impact of PMRT on types of immediate BRR showed its potential feasibility in this setting, with lower morbidity rates compared with those of implant‐based procedures^5^, ^16^, ^17^, ^18^. Surprisingly, the rapid adoption of immediate implant‐based reconstruction in about 70 per cent of women, compared with 34 per cent of autologous procedures when PMRT is recommended, may be influenced by surgeon and patient preferences, regardless of current evidence^15^, ^17^, ^19^.

Increasing recommendations for PMRT and immediate BRR have prompted a need to consider their optimal sequence. Previous systematic reviews have not provided clarity concerning the choice between immediate and delayed BRR^9^. Despite this, immediate autologous BRR is commonly recommended in the setting of PMRT, given the potential long‐term benefits on patients' QOL and breast cosmetic satisfaction^20^, ^21^. Currently, immediate autologous BRR and PMRT recommendations are variable^22^, ^23^. A systematic review^24^ in 2011 showed methodological variations in the definitions of surgical complications, precluding interstudy comparisons.

Complications of autologous breast reconstruction with PMRT include: poor wound‐healing, flap‐related fat necrosis, fibrosis and contracture, which reduce breast volume^5^. Surgical complications contribute variably to decreased patient satisfaction and impaired cosmetic outcomes^5^. A standardized core set of outcomes for BRR has been proposed^25^ involving a range of complications, including flap‐related complications and the need for further unplanned surgery. The BRR core outcome set has yet to recommend a standardized measurement tool for evaluating surgical complications. Most surgeons use the Clavien–Dindo classification (CDC)^26^. Patient‐reported QOL outcomes using validated BRR questionnaires, such as the BREAST‐Q and the European Organisation for Research and Treatment of Cancer (EORTC) Quality‐of‐Life Questionnaire (QLQ)‐BRECON23, are recommended to evaluate comparative effectiveness^20^, ^27^, ^28^, ^29^, ^30^, ^31^, ^32^.

This systematic review aimed to evaluate the quality and strengths of the current evidence regarding surgical complications in autologous abdominal flaps in the context of the receipt and timing of radiotherapy related to PMRT^5^, ^6^ and, less commonly, neoadjuvant radiotherapy, generally administered before skin‐sparing mastectomy and immediate breast reconstruction^33^, including assessment of QOL^34^.

## Methods

The protocol was registered and published on the Prospective Register of Systematic Reviews PROSPERO (CRD42017077945)^35^. The authors adhered to the PRISMA statement^36^.

### Search strategies

A comprehensive search of the MEDLINE (Ovid SP), Embase (Ovid SP), Google Scholar, Cochrane Controlled Register of Trials (CENTRAL), Science citation index databases and http://clinicaltrials.gov (January 2000 to August 2018) was conducted, identifying the relevant studies. Combinations of Medical Subject Headings (MeSH) terms and free text were used, including Boolean logical operators for the search strategy. References of included articles were also screened for their relevance. The example of an Embase (Ovid SP) search strategy was adopted for other databases (*Appendix S1*, supporting information).

### Identification and selection of studies

Database‐related searches were entered into an EndNote™ X8 library (Clarivate Analytics, Philadelphia, Pennsylvania, USA). Study screening was performed independently in two stages by two investigators using prespecified screening criteria.

In stage 1, two authors independently screened titles and abstracts. Discrepancies were resolved by consensus with the senior author. Remaining doubts regarding an article resulted in a review of the complete publication.

In stage 2, full‐text studies from stage 1 were screened independently for their eligibility by two reviewers. Discrepancies were resolved by consensus with a third reviewer. Authors of eligible studies were contacted (via e‐mail) to reconcile any methodological issues or to provide more detailed information on data for individual types of autologous flap.

### Study design

All primary human studies evaluating surgical complications for autologous free flap (microvascular) abdominal BRR in breast cancer and types of radiotherapy (PMRT, neoadjuvant and no radiotherapy) were included. Outcomes also included patient‐reported QOL and cosmetic assessments. Radiotherapy groups were compared with a control or no radiotherapy group in comparative studies, compatible with immediate and delayed BRR. Commonly performed autologous abdominal flaps included: deep inferior epigastric perforator (DIEP), transverse rectus abdominis myocutaneous (TRAM) and the superficial inferior epigastric artery perforator (SIEA)^6^.

### Inclusion criteria

Inclusion criteria were: women aged at least 18 years with a diagnosis of invasive breast cancer (TNM categories: T0–3, N1–3, Mx, M0), undergoing immediate or delayed abdominal autologous BRR using free flaps (DIEP, TRAM or SIEA) who received adjuvant radiotherapy (PMRT), neoadjuvant radiotherapy or no radiotherapy.

Clinical studies that involved at least 50 patients were included (RCTs, prospective and retrospective comparative observational studies, and case series).

### Exclusion criteria

Review articles, conference abstracts, simulation studies and clinical studies in non‐human subjects were not included, along with studies involving patients who received segmental or partial mastectomy, technical descriptions of operative repair with no outcome measures, BRR unrelated to breast cancer, implant‐based reconstructions and other non‐abdominal autologous flaps.

### Risk of bias and quality of studies

Cochrane's ROBINS‐I (Risk Of Bias In Non‐randomised Studies – of Interventions) tool was used for comparative studies^37^. This comprises seven domains from which the risk of bias may be ascertained to produce an overall risk‐of‐bias score^37^. The Grading of Recommendations, Assessment, Development, and Evaluations (GRADE) tool^38^ was used to evaluate the methodological quality of individual studies.

### Study outcomes

Primary outcomes were surgical complications including: Clavien–Dindo classification (CDC) grades II and III^26^; partial flap loss; total flap loss; fat necrosis (CDC grades, when reported)^39^; number(s) of unplanned reoperations for surgical complications (excluding cosmetic revisions); and number(s) of total complications. A surgical complication was defined as an adverse, postoperative, surgery‐related event that required additional treatment^16^. If CDC grades were not defined, the complications reported by the included studies were graded retrospectively according to the CDC by two independent authors; any discrepancy was discussed and agreed with the senior author.

Secondary outcomes were assessed using patient‐reported QOL‐validated questionnaires (COnsensus‐based Standards for the Selection of health Measurement INstruments (COSMIN)^40^, ^41^, Breast Questionnaire (BREAST‐Q), the EORTC Quality‐of‐Life Questionnaire (QLQ) – Breast Cancer 23^42^, the Quality‐of‐Life Cancer Generic Questionnaire (QLQ‐C30)^43^, the Numerical Pain Rating Scale (NPRS)^44^, ^45^, the Patient‐Reported Outcomes Measurement Information System – Profile 29 (PROMIS‐29)^46^, the McGill Pain Questionnaire (MPQ)^47^, the Generalized Anxiety Disorder Scale (GAD‐7)^48^ and the Patient Health Questionnaire (PHQ‐9)^49^), as well as assessment of cosmetic outcomes using independent panel or self assessments of medical photographs, and surface imaging using the Vectra® XT three‐dimensional system^50^ (Canfield Scientific, Parsippany, New Jersey, USA).

### Data extraction, collection and management

Two authors independently extracted data from full‐text articles using a standard data form. Any discrepancies were resolved by consensus with a third reviewer. Reporting authors of original articles were contacted on up to two occasions relating to missing data or where additional information was required.

Data extraction included: first author, year of publication, study design, study setting, number of centres, duration of follow‐up, study population and participant demographics (mean age, BMI, smoking, co‐morbidities).

Surgical complications were recorded using CDC: grades II–III^26^. Two authors reviewed eligible studies and classified each complication according to the CDC^26^ if unreported.

QOL and cosmetic outcomes were listed.

### Statistical analysis

When two or more studies reported outcome data, these were pooled using Review Manager 5.3 software (The Cochrane Collaboration, The Nordic Cochrane Centre, Copenhagen, Denmark). Odds ratios with 95 per cent confidence intervals were used to evaluate dichotomous outcomes (surgical complications). Standard mean differences (with 95 per cent c.i.) were used for continuous outcomes between treatment groups. Rates of each complication (fat necrosis, partial and total flap loss, infection and wound complications (dehiscence and delayed wound healing)) were compared for PMRT (*versus* no radiotherapy) and neoadjuvant radiotherapy (*versus* no radiotherapy). Data were also pooled to provide an overall summary measure of combined radiotherapy (adjuvant and neoadjuvant) compared with no radiotherapy.

Heterogeneity between studies^51^ was assessed in Review Manager 5.3 using the Higgins and Thompson *I* ^2^ statistic^52^. Levels of heterogeneity were defined as: low (*I* ^2^ less than 50 per cent), moderate (*I* ^2^ = 50–80 per cent) and high (*I* ^2^ above 80 per cent). A random‐effects model was used for cohorts with heterogeneity (*I* ^2^ above 50 per cent)^53^. As heterogeneity was generally moderate or high, and outcome measures differed between studies, these were combined using the DerSimonian and Laird random‐effects model. Results of meta‐analyses are shown as forest plots. A sensitivity analysis was performed where possible, to evaluate whether outcomes differed when restricting the analysis exclusively to high‐quality studies.

Clinically meaningful differences in QOL items/questions or domain scores may vary depending on response shift, that is a change in the meaning of QOL scores over time^54^. This is relevant in longitudinal studies and may influence clinical significance, defined as greater than 5‐point score differences for EORTC QLQ‐C30 and QLQ‐BR23^42^, ^43^, ^54^. Clinically meaningful differences are currently being evaluated using a number of methods such as qualitative interviews and using predefined clinical anchors^55^. Clinically meaningful differences in QOL should be differentiated from statistical significance^55^. BREAST‐Q findings have been compared with large population‐derived normative data, facilitating clinically meaningful interpretation of data^56^, ^57^.

## Results

**Figure 1:**
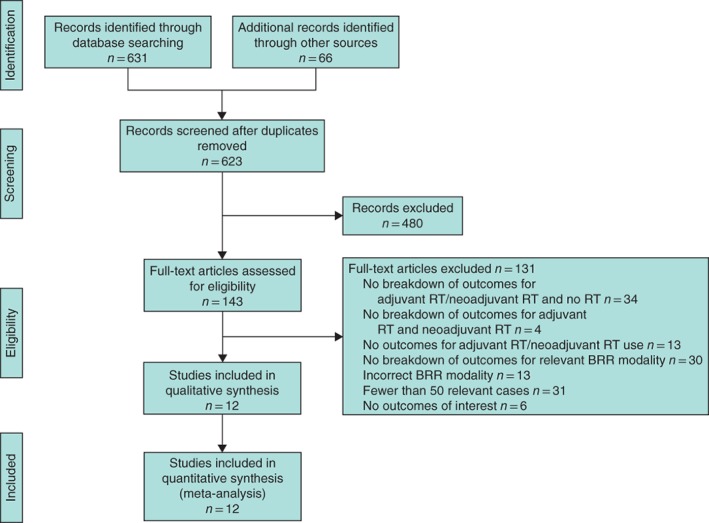
PRISMA diagram for the review RT, radiotherapy; BRR, breast reconstruction.

A total of 697 studies were identified. Of these, 12 studies^58^, ^59^, ^60^, ^61^, ^62^, ^63^, ^64^, ^65^, ^66^, ^67^, ^68^, ^69^ (including 1756 patients) evaluated adjuvant radiotherapy (350 patients), neoadjuvant radiotherapy (723) and no radiotherapy (683) (*Fig*. 1). There were three prospective study designs^59^, ^60^, ^62^ and nine that were retrospective^58^, ^61^, ^63^, ^64^, ^65^, ^66^, ^67^, ^68^, ^69^, but no RCTs. There were two multicentre (1 prospective^62^ and 1 retrospective^66^) and ten single‐centre studies (2 prospective^59^, ^60^ and 8 retrospective^58^, ^61^, ^63^, ^64^, ^65^, ^67^, ^68^, ^69^) (*Table* 1). Study quality (GRADE) was low in eight studies^58^, ^59^, ^61^, ^63^, ^64^, ^65^, ^66^, ^68^ and moderate in the other four^60^, ^62^, ^67^, ^69^, with an overall high risk of bias. A summary of baseline characteristics, including numbers of centres, country of origin, dates, patient numbers, breast cancer pathology and adjuvant medical treatments in comparative adjuvant and neoadjuvant radiotherapy groups, including non‐comparative studies, is provided in *Table* *S1* (supporting information).

**Table 1:** Study summaries: comparative adjuvant or neoadjuvant radiotherapy in autologous breast reconstruction, and non‐comparative studies (adjuvant radiotherapy or neoadjuvant radiotherapy only)

Reference	Years	Country	No. of centres	Type of BRR flap	Overall follow‐up (months)	Group differences in baseline characteristics¶	RT dose and regimen
Baumann *et al*.^69^, ‡	2005–2009	USA	1	msTRAM; DIEP; SIEA	11*	n.a.	Total 60 Gy; missing details
Billig *et al*.^62^, §	2012–2017	USA and Canada	11	TRAM; DIEP; SIEA	24	Adjuvant RT: more non‐Hispanic patients (*P* = 0·001), bilateral BRR (*P* = 0·002), DIEP/SIEA (*P* < 0·001), adjuvant chemotherapy (*P* < 0·001); less TRAM (*P* < 0·001)#	Total 50·4 Gy over 4 weeks, daily (28 fractions of 1·8 Gy)
Chatterjee *et al*.^59^, §	1995–2005	UK	1	DIEP	42 (12–120)†	Adjuvant RT: more IDC (*P* = 0·02), LVI (*P* = 0·044), positive axillary LN (*P* < 0·001)	Total 45 Gy over 4 weeks (20 fractions)
Cooke *et al*.^60^, §	2012–2015	Canada	1	DIEP; SIEA	12	Adjuvant RT: higher TNM staging, positive LN, more chemotherapy (*P* values not provided)	Total 50/50·4 Gy over 4 weeks, daily (25 fractions of 2 Gy/28 fractions of 1·8 Gy)
Huang *et al*.^63^, ‡	1997–2001	Taiwan	1	TRAM	40 (24–74)†	n.a.	Total 50 Gy; missing details
Levine *et al*.^67^, ‡	1999–2011	USA	1	msTRAM; DIEP; SIEA	22·7*	n.a.	Missing details
Modarressi *et al*.^64^, ‡	2007–2013	Switzerland	1	DIEP	1	n.a.	Missing details
Mull *et al*.^65^, ‡	2003–2014	USA	1	msTRAM; TRAM; DIEP	1	Neoadjuvant RT: more chemotherapy (*P* < 0·01), higher TNM staging (*P* < 0·01); less hypertension/CAD (*P* = 0·03)	Missing details
O'Connell *et al*.^58^, ‡	2009–2014	UK	1	DIEP	44·3 (i.q.r. 31·1–56·4)†	Adjuvant and neoadjuvant RT: more chemotherapy and endocrine therapy as less DCIS/less advanced invasive disease (*P* values not provided)	Total 40 Gy over 3 weeks (15 fractions)
Peeters *et al*.^66^, ‡	1997–2003	Belgium	2	DIEP	≥ 12	n.a.	Total 50 Gy; missing details
Rogers and Allen^61^, ‡	1994–1999	USA	1	DIEP	18·7*	n.a.	Total 50·5 Gy over 6·5 weeks (missing details)
Temple *et al*.^68^, ‡	1990–2001	USA	1	TRAM	≥ 12	n.a.	Total 58 Gy; missing details

Values are *mean and †median (range), unless indicated otherwise. ‡Retrospective study; §prospective study. ¶Radiotherapy (RT) *versus* no RT, except #group difference values are for adjuvant RT *versus* neoadjuvant RT. BRR, breast reconstruction; (ms)TRAM, (muscle‐sparing) transverse rectus abdominis myocutaneous; DIEP, deep inferior epigastric artery perforator; SIEA, superficial inferior epigastric artery perforator; IDC, invasive ductal carcinoma; LVI, lymphovascular invasion; LN, lymph node; n.a., not applicable/available; CAD, coronary artery disease; DCIS, ductal carcinoma *in situ*.

### Clinical outcomes (*Tables* 2, 3, 4, 5)

**Table 2:** Surgical complications: immediate autologous breast reconstruction and adjuvant radiotherapy including non‐comparative studies (adjuvant radiotherapy only)

			No. of patients		Follow‐up (months)		Total no. of complications		No. of reoperations for complications	
Reference	GRADE	ROBINS‐I	Adjuvant RT	No adjuvant RT	Adjuvant RT	No adjuvant RT	Adjuvant RT	No adjuvant RT	Adjuvant RT	No adjuvant RT
Chatterjee *et al*.^59^	Low	Serious	22	46	54*	36*	n.a.	n.a.	n.a.	n.a.
Cooke *et al*.^60^	Moderate	Moderate	64	61	12	12	20	16	6	1
O'Connell *et al*.^58^	Low	Serious	28	80	27·5*	48·7*	11	20	4	8
Peeters *et al*.^66^	Low	Serious	16	109	≥ 12	≥ 12	n.a.	n.a.	n.a.	n.a.
Rogers and Allen^61^	Low	Serious	30	30	19·9	17·4	65	41	32	26
Billig *et al*.^62^	Moderate	Moderate	108	n.a.	24	n.a.	81	n.a.	5	n.a.
Huang *et al*.^63^	Low	Serious	82	n.a.	40*	n.a.	131	n.a.	5	n.a.

* Values are median. GRADE, Grading of Recommendation, Assessment, Development, and Evaluation (tool for grading the quality of evidence); ROBINS‐I, Risk Of Bias In Non‐randomised Studies – of Interventions (tool for assessing risk of bias); RT, radiotherapy; n.a., not applicable/available.

**Table 3:** Clavien–Dindo classification of surgical complications: immediate autologous breast reconstruction and adjuvant radiotherapy including non‐comparative studies (adjuvant radiotherapy only)

	Adjuvant RT *versus* no adjuvant RT						
					Clavien‐Dindo complication grade†		
Reference	Total flap loss	Partial flap loss*	Fat necrosis*	Wound dehiscence and delayed wound healing*	II	IIIa	IIIb
Chatterjee *et al*.^59^	n.a.	n.a.	n.a.	n.a.	n.a.	n.a.	n.a.
Cooke *et al*.^60^	0 *versus* 0	9 *versus* 6	2 *versus* 1	3 *versus* 5	2 *versus* 4	n.a.	6 *versus* 1
O'Connell *et al*.^58^	0 *versus* 0	0 *versus* 0	1 *versus* 2	4 *versus* 9	3 *versus* 3	3 *versus* 3	1 *versus* 5
Peeters *et al*.^66^	n.a.	n.a.	6 *versus* 36	n.a.	n.a.	n.a.	n.a.
Rogers and Allen^61^	n.a.	n.a.	7 *versus* 0‡	11 *versus* 8	5 *versus* 7	7 *versus* 0	25 *versus* 26
Billig *et al*.^62^	0 *versus* n.a.	n.a.	4 *versus* n.a.	17 *versus* n.a.	8 *versus* n.a.	n.a.	5 *versus* n.a.
Huang *et al*.^63^	0 *versus* n.a.	n.a.	7 *versus* n.a.	n.a.	82 *versus* n.a.	5 *versus* n.a.	n.a.

*Complication grades were not always defined or classified. †Grade II, complications requiring pharmacological treatment with drugs other than those allowed for grade I complications (drugs other than antiemetics, antipyretics, analgesics, diuretics and electrolytes); grade IIIa, complications requiring surgical intervention not under general anaesthesia; grade IIIb, complications requiring surgical intervention under general anaesthesia. RT, radiotherapy; n.a. not applicable/available. ‡*P* < 0·050.

**Table 4:** Surgical complications: delayed autologous breast reconstruction and neoadjuvant radiotherapy including non‐comparative studies (neoadjuvant radiotherapy only)

			No. of patients		Follow‐up (months)		Total no. of complications		No. of reoperations for complications	
Reference	GRADE	ROBINS‐I	Neoadjuvant RT	No neoadjuvant RT	Neoadjuvant RT	No neoadjuvant RT	Neoadjuvant RT	No neoadjuvant RT	Neoadjuvant RT	No neoadjuvant RT
Modarressi *et al*.^64^	Low	Serious	60	45	1	1	20	9	n.a.	n.a.
Mull *et al*.^65^	Low	Serious	142	312	1	1	26	45	26	45
O'Connell *et al*.^58^	Low	Serious	38	80	50·3*	48·7*	12	20	3	8
Peeters *et al*.^66^	Low	Serious	77	109	≥ 12	≥ 12	n.a.	n.a.	n.a.	n.a.
Baumann *et al*.^69^	Moderate	Moderate	189	n.a.	11†	n.a.	88	n.a.	69	n.a.
Billig *et al*.^62^	Moderate	Moderate	67	n.a.	24	n.a.	37	n.a.	1	n.a.
Levine *et al*.^67^	Moderate	Moderate	50	n.a.	22·7†	n.a.	n.a.	n.a.	3	n.a.
Temple *et al*.^68^	Low	Serious	100	n.a.	≥ 12	n.a.	41	n.a.	18	n.a.

Values are *median and †mean. GRADE, Grading of Recommendation, Assessment, Development, and Evaluation (tool for grading the quality of evidence); ROBINS‐I, Risk Of Bias In Non‐randomised Studies – of Interventions (tool for assessing risk of bias); RT, radiotherapy; n.a., not applicable/available.

**Table 5:** Clavien–Dindo classification of surgical complications: delayed autologous breast reconstruction and neoadjuvant radiotherapy including non‐comparative studies (neoadjuvant radiotherapy only)

	Neoadjuvant RT *versus* no neoadjuvant RT						
					Clavien‐Dindo complication grade†		
Reference	Total flap loss	Partial flap loss*	Fat necrosis*	Wound dehiscence and delayed wound healing*	II	IIIa	IIIb
Modarressi *et al*.^64^	2 *versus* 1	12 *versus* 2	n.a.	n.a.	n.a.	n.a.	n.a.
Mull *et al*.^65^	5 *versus* 15	7 *versus* 5‡	n.a.	n.a.	n.a.	n.a.	26 *versus* 45
O'Connell *et al*.^58^	0 *versus* 0	0 *versus* 0	2 *versus* 2	7 *versus* 9	2 *versus* 3	0 *versus* 3	3 *versus* 5
Peeters *et al*.^66^	n.a.	n.a.	29 *versus* 36	n.a.	n.a.	n.a.	n.a.
Baumann *et al*.^69^	5 *versus* n.a.	14 *versus* n.a.	15 *versus* n.a.	22 *versus* n.a.	4 *versus* n.a.	n.a.	69 *versus* n.a.
Billig *et al*.^62^	0 *versus* n.a.	n.a.	7 *versus* n.a.	11 *versus* n.a.	4 *versus* n.a.	n.a.	1 *versus* n.a.
Levine *et al*.^67^	n.a.	1 *versus* n.a.	n.a.	n.a.	n.a.	n.a.	n.a.
Temple *et al*.^68^	2 *versus* n.a.	7 *versus* n.a.	16 *versus* n.a.	n.a.	n.a.	n.a.	18 *versus* n.a.

* Complication grades were not always defined or classified.

† Grade II, complications requiring pharmacological treatment with drugs other than those allowed for grade I complications (drugs other than antiemetics, antipyretics, analgesics, diuretics and electrolytes); grade IIIa, complications requiring surgical intervention not under general anaesthesia; grade IIIb, complications requiring surgical intervention under general anaesthesia. RT, radiotherapy; n.a. not applicable/available.

‡ *P* < 0·050.

No study prospectively graded surgical complications according to an accepted classification such as CDC (fat necrosis, partial or total flap loss, infection and wound complications). One study^64^ graded partial flap loss using a novel flap necrosis classification system, adapted from Kwok *et al*.^70^. Only 30 per cent of all surgical complications (30 of 99) reported across the 12 included studies were defined *a priori*.

### Adjuvant post‐mastectomy radiotherapy

**Figure 2:**
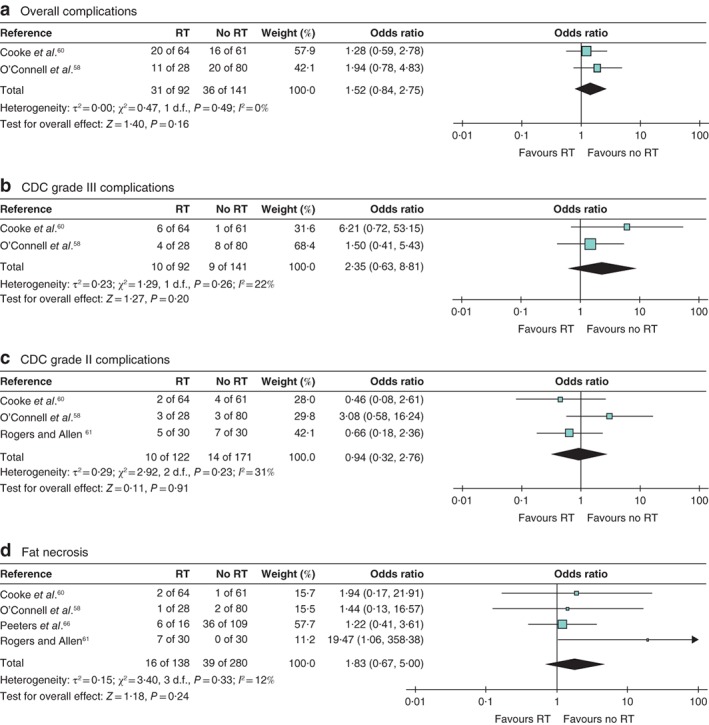
Forest plots comparing adjuvant radiotherapy with no radiotherapy **a** Overall complications, **b** Clavien–Dindo classification (CDC) grade III complications, **c** CDC grade II complications, **d** fat necrosis. A Mantel–Haenszel random‐effects model was used for meta‐analysis. Odds ratios are shown with 95 per cent confidence intervals. RT, radiotherapy.

Meta‐analyses comparing PMRT (350 patients; mean follow‐up 27·1 (range 12·0–54·0) months) and no radiotherapy (326 patients; mean follow‐up 25·2 (12·0–48·7) months) showed no interstudy differences in rates of: overall complications (233 patients; odds ratio (OR) 1·52 (95 per cent c.i. 0·84 to 2·75), *Z* = 1·40, *P* = 0·160) (*Fig*. 2 *a*); CDC grade III surgical complications (233 patients; OR 2·35 (0·63 to 8·81), *Z* = 1·27, *P* = 0·200) (*Fig*. 2 *b*); CDC grade II (293 patients; OR 0·94 (0·32 to 2·76), *Z* = 0·11, *P* = 0·910) (*Fig*. 2 *c*); or fat necrosis (418 patients; OR 1·83 (0·67 to 5·00), *Z* = 1·18, *P* = 0·240) (*Fig*. 2 *d*). There were no differences in rates of infection (293 patients; OR 0·94 (0·32 to 2·76), *Z* = 0·11, *P* = 0·910) (*Fig*. *S1* *a*, supporting information) or wound complications (293 patients; OR 1·16 (0·56 to 2·39), *Z* = 0·40, *P* = 0·690) (*Fig*. *S1* *b*, supporting information). There were no total flap losses.

### Neoadjuvant radiotherapy

**Figure 3:**
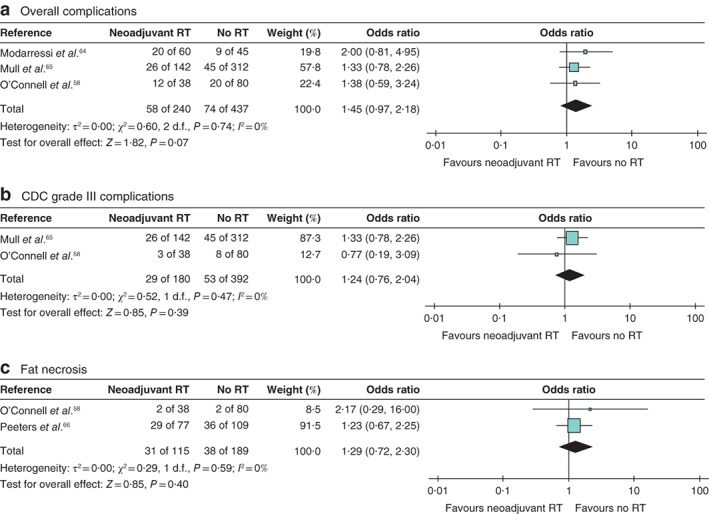
Forest plot comparing neoadjuvant radiotherapy with no radiotherapy **a** Overall complications, **b** Clavien–Dindo classification (CDC) grade III complications, **c** fat necrosis. A Mantel–Haenszel random‐effects model was used for meta‐analysis. Odds ratios are shown with 95 per cent confidence intervals. RT, radiotherapy.

Comparisons between neoadjuvant radiotherapy (723 patients; mean follow‐up 16·8 (range 1·0–50·3) months) and no radiotherapy (546 patients; mean follow‐up 15·7 (1·0–48·7) months) showed no differences in overall complications (677 patients; OR 1·45 (95 per cent c.i. 0·97 to 2·18), *Z* = 1·82, *P* = 0·070) (*Fig*. 3 *a*) and CDC grade III surgical complications (572 patients; OR 1·24 (0·76 to 2·04), *Z* = 0·85, *P* = 0·390) (*Fig*. 3 *b*). One comparative study^58^ reported similar CDC grade II complications between neoadjuvant and no radiotherapy (118 patients; OR 1·43 (0·23 to 8·91), *Z* = 0·38, *P* = 0·700). There were no differences in rates of fat necrosis (304 patients; OR 1·29 (0·72 to 2·30), *Z* = 0·85, *P* = 0·400) (*Fig*. 3 *c*). Rates of partial flap loss were higher for neoadjuvant radiotherapy than for no radiotherapy (559 patients; OR 3·85 (1·51 to 9·76), *Z* = 2·83, *P* = 0·005) (*Fig*. *S2* *a*, supporting information), with no differences in rates of total flap loss (559 patients; OR 0·81 (0·31 to 2·09), *Z* = 0·44, *P* = 0·660) (*Fig*. *S2* *b*, supporting information).

### Combined adjuvant and neoadjuvant radiotherapy

**Figure 4:**
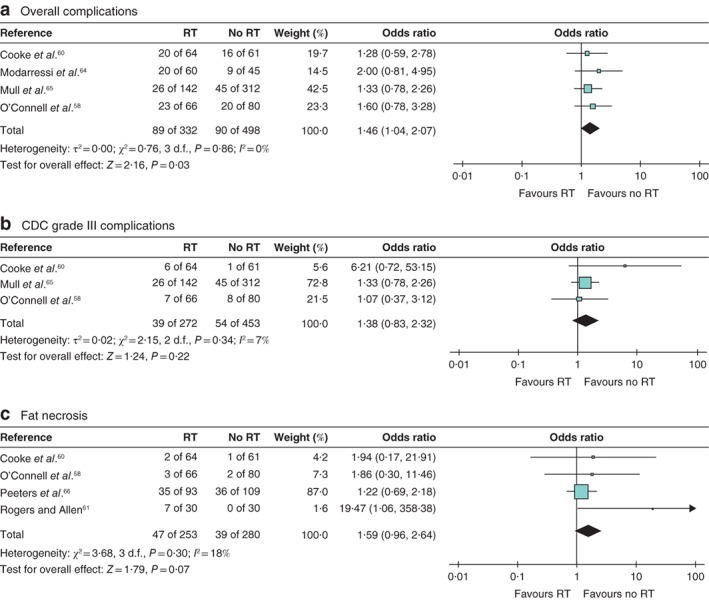
Forest plot comparing combined adjuvant and neoadjuvant radiotherapy with no radiotherapy **a** Overall complications, **b** Clavien–Dindo classification (CDC) grade III complications, **c** fat necrosis. A Mantel–Haenszel random‐effects model was used for meta‐analysis. Odds ratios are shown with 95 per cent confidence intervals. RT, radiotherapy.

Meta‐analyses of pooled PMRT and neoadjuvant radiotherapy compared with pooled no radiotherapy groups (mean follow‐up 18·3 (range 1·0–48·7) months) were performed as a potential hypothesis‐generating exercise. This showed significantly higher overall complications in the combined radiotherapy groups compared with no radiotherapy (830 patients; OR 1·46 (95 per cent c.i. 1·04 to 2·07), *Z* = 2·16, *P* = 0·030) (*Fig*. 4 *a*). There were no interstudy differences in: CDC grade III complications (725 patients; OR 1·38 (0·83 to 2·32), *Z* = 1·24, *P* = 0·220) (*Fig*. 4 *b*); CDC grade II complications (331 patients; OR 0·89 (0·37 to 2·10), *Z* = 0·28, *P* = 0·780) (*Fig*. *S3* *a*, supporting information); rates of fat necrosis (533 patients; OR 1·59 (0·96 to 2·64), *Z* = 1·79, *P* = 0·070) (*Fig*.  4 *c*); or emergency reoperations for complications (725 patients; OR 1·38 (0·83 to 2·32), *Z* = 1·24, *P* = 0·220) (*Fig*. *S3* *b*, supporting information). Rates of partial flap loss were also higher in the combined *versus* no radiotherapy groups (684 patients; OR 2·59 (1·27 to 5·28), *Z* = 2·63, *P* = 0·009) (*Fig*. *S3* *c*, supporting information), with no differences in rates of total flap loss (559 patients; OR 0·81 (0·31 to 2·09), *Z* = 0·44, *P* = 0·660) (*Fig*. *S3* *d*, supporting information), infection (331 patients; OR 0·89 (0·37 to 2·10), *Z* = 0·28, *P* = 0·780) (*Fig*. *S3* *e*, supporting information) or wound complications (dehiscence/delayed wound healing) (331 patients; OR 1·29 (0·68 to 2·47), *Z* = 0·78, *P* = 0·430) (*Fig*. *S3* *f*, supporting 1information).

### Assessment of heterogeneity and meta‐analyses

Clinical outcomes within studies of PMRT *versus* no radiotherapy were homogeneous (*I* ^2^ values below 50 per cent). All remaining meta‐analyses of outcomes were similar (neoadjuvant radiotherapy *versus* no radiotherapy, pooled PMRT and neoadjuvant radiotherapy *versus* no radiotherapy).

### Quality of life

There was limited reporting of patient‐reported QOL; outcomes were detailed in only two prospective studies^60^, ^62^ and one retrospective study^58^, with small patient numbers and short follow‐ups for the PMRT groups^58^, ^60^, ^62^. *A priori* hypothesis‐driven selection of QOL domains was absent from methods^58^, ^60^, ^62^, with no reporting of missing data or how this problem was tackled^34^.

Three studies^58^, ^60^, ^62^ used the BREAST‐Q and one^60^ used the breast cancer‐specific questionnaire (EORTC QLQ‐BR23)^42^. One small study^58^ reported significantly better ‘satisfaction with breast’ (*P* = 0·008) after a median follow‐up of 27·5 months for PMRT compared with 48·7 months for no radiotherapy (*Table* *S2*, supporting information). The moderate‐quality comparative prospective study^60^ found a significant adverse impact of PMRT on breast symptoms at 1 year (*P* < 0·001) compared with no radiotherapy (*Table* *S2*, supporting information).

The third study^62^ evaluated serial QOL outcomes, concluding a significant impact of PMRT on QOL domains (BREAST‐Q) at 1 and 2 years, despite the absence of a control group (no radiotherapy). Moreover, clinical significance was defined as *P* = 0·05, which may not account for multiple variables (*Table* *S2*, supporting information)^43^, ^62^. Highly significant abdominal adverse effects in a small patient group (108 patients) may be unrelated to PMRT, but rather an indication of donor site morbidity. Interestingly, when evaluating the impact of neoadjuvant radiotherapy in a small non‐comparative study^62^, significant time‐related improvements in most QOL domains were observed, except lower physical well‐being relating to the abdomen at 1 year (*Table* *S3*, supporting information).

### Cosmetic outcomes

Three studies^58^, ^61^, ^63^ evaluated PMRT and the effects on aesthetic outcomes (187 patients). There was no standardized evaluation of cosmetic outcomes, precluding meta‐analyses. Studies lacked robust methodology.

## Discussion

The mixture of underpowered observational studies included in this review were, in large part, lacking contemporaneous data to reflect current practice. Most were retrospective single‐centre cohorts, demonstrating poor levels of clinical evidence (levels 3 and 4) with insufficient follow‐up^11^.

A previous study^24^ of over 40 000 women undergoing BRR in 134 studies found that only 20 per cent reported *a priori* surgical complications, as well as inconsistent interstudy definitions^24^. The present review found similar interstudy discrepancies, without uniform adoption of the CDC^26^. The present authors graded all reported surgical complications using the CDC. All surgical interventions were graded as CDC IIIa or IIIb, and surgical reoperations were differentiated according to whether they were for complications or cosmetic revisions. Some complications were not amenable to retrospective grading in three studies^64^, ^66^, ^67^. In one^66^, it was not possible to determine whether fat necrosis required surgical revision for each radiotherapy group (adjuvant or neoadjuvant), compared with no radiotherapy. A second^64^ omitted individual abdominal complications relative to timings of radiotherapy, and the third^67^ omitted overall numbers of complications. Reviewed studies also failed to define postoperative wound infections according to Centers for Disease Control and Prevention criteria^71^.

The IDEAL (Idea, Development, Exploration, Assessment, Long‐term study) Collaboration describes key methodological criteria for robust prospective cohort studies^72^: studies should be powered on the effect size of primary outcomes evaluating interventions of interest. The Mastectomy and Breast Reconstruction Outcomes Collaborative (MROC) is a multicentre prospective cohort study that provides IDEAL level 2b evidence for clinical safety and satisfactory QOL outcomes in the evaluation of surgical complications in immediate autologous reconstructions with PMRT *versus* no radiotherapy (delayed BRR) in 11 US centres^17^, ^60^. The MROC cohort data were excluded from this systematic review based on its reporting of group‐related summative data for all types of autologous reconstruction, as opposed to individual abdominal donor sites.

The MROC has reported all surgical complications at 2 years and demonstrated that PMRT (*versus* no radiotherapy) was significantly associated with a greater risk of developing any complication (OR 1·50 (95 per cent c.i. 1·20 to 1·86); *P* < 0·001), reoperative complications (OR 1·52 (1·17 to 1·97); *P* < 0·002) and wound infection (OR 2·77 (1·78 to 4·31); *P* < 0·001)^16^. Autologous BRR was done more commonly in irradiated than non‐irradiated patients (38 *versus* 25 per cent respectively; *P* < 0·001), with similarly low rates (1–2·4 per cent) of reconstruction failure at 2 years^17^.

Eligible studies in the present systematic review were significantly underpowered in comparison with the MROC study, which evaluated irradiated autologous BRR at 1 year (236 patients) and 2 years (199), and non‐irradiated procedures at 1 year (1625) and 2 years (332). The MROC data showed no differences between radiotherapy and no radiotherapy groups in the rates of total complications (25·6 *versus* 28·3 per cent respectively), major complications (17·6 *versus* 22·9 per cent) or flap failure (1·0 *versus* 2·4 per cent) at 2 years after immediate autologous reconstruction^17^. Studies in the present review showed significantly lower rates of major complications after radiotherapy compared with the MROC results, suggesting suboptimal overall reporting of surgical complications in the reviewed studies^24^.

The retrospective grading of surgical complications in the two moderate‐quality studies reported showed a rate of major complications (CDC grade IIIb) of 9 per cent (6 of 64) at 1 year, and 4·6 per cent (5 of 108) at 2 years^60^, ^62^. These rates are also likely to reflect under‐reporting compared with the MROC rates of 14·8 per cent (35 of 236) at 1 year and 17·6 per cent (35 of 199) at 2 years^17^. Despite its strengths, the MROC cohort is based on the review of complications from electronic patient records, potentially also underestimating true complication rates^17^.

One way to measure what matters to patients is to use patient‐reported outcome measures (PROMs) to assess the effects of disease or treatment on symptoms, functioning and health‐related QOL^34^. In this systematic review, PROMs were poorly reported and underpowered for overall small effect sizes of individual QOL domains^43^. Preliminary conclusions regarding statistical significance were not substantiated by adequate patient numbers, lack of a comparator group or prospectively defined time points for questionnaire collection^58^. Standardized and objective evaluations of cosmetic outcome have also remained elusive with emerging adoption of newer technologies such as the Vectra® XT^58^. Robust study designs evaluating these innovations should be accompanied by surgery‐ and disease‐specific questionnaires^34^.

Clear recommendations for the optimal timing of radiotherapy in relation to autologous BRR will remain elusive until information from high‐quality systematic reviews forms part of shared preoperative decision‐making^73^.

Adequately powered prospective studies and ongoing audits, to allow comparisons of postoperative radiotherapy with neoadjuvant radiotherapy, are warranted. Current evidence for irradiating autologous abdominal flaps remains weak, involving only two moderate‐quality studies of the 12 included in this report. Future cohort studies should be designed and powered to take advantage of newly evolving study designs, such as multiple‐cohort RCTs or trials within cohorts^74^. These designs permit collection of big data within registry or cohort platforms, and allow multiple synchronous randomized trials to be conducted in a cost‐effective manner^74^.

## Supporting information


**Table S1** Baseline characteristics of eligible studies
**Table S2** Study evaluating patient‐reported quality of life in immediate autologous breast reconstruction comparing adjuvant radiotherapy with no radiotherapy, and non‐comparative study (adjuvant radiotherapy only)
**Table S3** Study evaluating patient‐reported quality of life in delayed autologous breast reconstruction comparing neoadjuvant radiotherapy with no radiotherapy, and non‐comparative study (neoadjuvant radiotherapy only)

**Fig. S1** Forest plot comparisons for adjuvant radiotherapy *versus* no adjuvant radiotherapy

**Fig. S2** Forest plot comparisons for neoadjuvant radiotherapy *versus* no neoadjuvant radiotherapy

**Fig. S3** Forest plot comparisons for combined adjuvant and neoadjuvant radiotherapy *versus* no radiotherapy
