# Supplementary material for: Immediate and delayed autologous abdominal microvascular flap breast reconstruction in patients receiving adjuvant, neoadjuvant or no radiotherapy: a meta‐analysis of clinical and quality‐of‐life outcomes
Source: BJS Open. 2019 Dec 29;4(2):182–96. doi: 10.1002/bjs5.50245 (PMC7093792; doi:10.1002/bjs5.50245)
Supplement: Supplementary file 1 — Table S1 Baseline characteristics of eligible studies Table S2 Study evaluating patient‐reported quality of life in immediate autologous breast reconstruction comparing adjuvant radiotherapy with no radiotherapy, and non‐comparative study (adjuvant radiotherapy only) Table S3 Study evaluating patient‐reported quality of life in delayed autologous breast reconstruction comparing neoadjuvant radiotherapy with no radiotherapy, and non‐comparative study (neoadjuvant radiotherapy only) [file BJS5-4-182-s001.docx]

**BJS5_50245**

**Immediate and delayed autologous abdominal microvascular flap breast reconstruction in patients receiving adjuvant, neoadjuvant or no radiotherapy: a meta-analysis of clinical and quality-of-life outcomes**

**A. Khajuria, W. N. Charles, M. Prokopenko, A. Beswick, A. L. Pusic, A. Mosahebi, D. J. Dodwell and Z. E. Winters**

**Appendix S1** Sample search strategy for Embase (OVID SP), with the adoption of a similar strategy for other databases

**‘Breast cancer’**

(1) exp Breast Neoplasms/ OR (breast* adj5 (neoplasm* or cancer* or tumor* or tumour* or carcinoma* or adenocarcinoma* or sarcoma* or dcis or ductal or infiltrate* or intraductal* or lobular or medullary or malignant* or reconstruct*))

**‘Autologous abdominal-based flaps’**

(2) exp surgical flaps OR deep inferior epigastric perforator flap/ OR DIEP flap* OR DIEAP flap* OR ((Deep and inferior and epigastric and perforator) adj2 flap*) OR Deep and inferior and epigastric and perforator and flap*) OR (rectus AND myocutaneous) OR (SIEA OR SIEAP) OR (Superficial and inferior and epigastric and perforator)

**‘Radiotherapy’**

(3) exp radiotherapy OR radiotherap* OR radiation OR irradiation OR brachytherap*

**‘Surgical complications’**

(4) exp complication OR surgical complication* OR Clavien-Dindo

**‘Patient-reported outcomes’**

(5) patient reported outcome measures OR (PROM or PROMs) OR quality of life OR ((patient* adj5 (report* or relate*) adj5 (outcome* or measure*)) OR (questionnaire* or interview* or self-report* or measure* or instrument* or scale* or tool* or construct*) OR (BREAST-Q or SV-POMS or Visual Analogue Scale or DAS-59 or SF-36 or HADS or Satisfaction Interview or Body Cathexis Scale or Rosenberg Self-Esteem or SASS or quality of Life Index or HAM-A or FACT-B or EORTC)

(1) AND (2) AND (3) AND (4) AND (5) AND (6)

Limit studies to those published between January 2000 and August 2018

DCIS, Ductal carcinoma *in situ*; BREAST-Q, Breast Questionnaire; SV-POMS, Shortened Version of the Profile of Mood States; DAS-59, The Derriford Appearance Scale; SF-36, Short Form Health Survey; HADS, Hospital Anxiety and Depression Score; SASS, Social Adaptation Self-evaluation Scale; HAM-A, Hamilton Anxiety Rating Scale; FACT-B, Functional Assessment of Cancer Therapy; EORTC, European Organization for Research and Treatment of Cancer.

**Table S1** Baseline characteristics of eligible studies

| **Study,**  **Dates,**  **Country, No. centre(s)** | **No. pts**  **Other treatments**  **Pathology** | | |
| --- | --- | --- | --- |
|  | **Adj RT** | **No RT** | **Neo RT** |
| **Baumann et al.^69^,**^‡^  **2005-2009**  **USA, 1 centre** | NA | NA | 189  Chemotherapy |
| **Billig et al.^62^,**^†^  **2012-2017**  **USA & Canada, 11 centres** | 108  Chemotherapy | NA | 67  Chemotherapy |
| **Chatterjee et al.^59^,**^†^  **1995-2005**  **UK, 1 centre** | 22  Chemotherapy, endocrine  DCIS 9%, IDC 73%, ILC 14% | 46  Chemotherapy, endocrine  DCIS 26%, IDC 41%, ILC 9% | NA |
| **Cooke et al.^60^,**^†^  **2012-2015**  **Canada, 1 centre** | 64  Chemotherapy, endocrine  DCIS 2% | 61  Chemotherapy, endocrine  DCIS 34%, CP 3% | NA |
| **Huang et al.^63^,**^‡^  **1997-2001**  **Taiwan, 1 centre** | 82  Chemotherapy, endocrine  IDC 90%, ILC 5%, MC 2% | NA | NA |
| **Levine et al.^67^,**^‡^  **1999-2011**  **USA, 1 centre** | NA | NA | 50 |
| **Modarressi et al.^64^,**^‡^  **2007-2013**  **Switzerland, 1 centre** | NA | 45 | 60 |
| **Mull et al.^65^,**^‡^  **2003-2014**  **USA, 1 centre** | NA | 312  Chemotherapy  DCIS/LCIS 19% | 142  Chemotherapy  DCIS/LCIS 8% |
| **O’Connell et al.^58^,**^‡^  **2009-2014**  **UK, 1 centre** | 28  Chemotherapy, endocrine | 80  Chemotherapy, endocrine | 38  Chemotherapy, endocrine |
| **Peeters et al.^66^,**^‡^  **1997-2003**  **Belgium, 2 centres** | 16 | 109 | 77 |
| **Rogers et al.^61^,**^‡^  **1994-1999**  **USA, 1 centre** | 30 | 30 | NA |
| **Temple et al.^68^,**^‡^  **1990-2001**  **USA, 1 centre** | NA | NA | 100 |

^†^Prospective studies.

^‡^Retrospective studies.

No., Number of; pts, Patients; Adj RT, Adjuvant Radiotherapy; RT, Radiotherapy; Neo RT, Neoadjuvant Radiotherapy; USA, United States of America; UK, United Kingdom; NA, Not Applicable/available; IDC, Invasive ductal carcinoma; DCIS, Ductal carcinoma *in situ*; ILC, Invasive lobular carcinoma; CP, Cystosarcoma phyllodes; MC, Medullary carcinoma; LCIS, Lobular carcinoma *in situ*.

**Table S2** Study evaluating patient-reported quality of life in immediate autologous breast reconstruction comparing adjuvant radiotherapy with no radiotherapy, and non-comparative study (adjuvant radiotherapy only)

| **Study,**  **Scoring description** | **BREAST-Q for Breast Reconstruction (BRR)** | | | | | | **EORTC QLQ-BR23 (100-point ordinal scale)** |
| --- | --- | --- | --- | --- | --- | --- | --- |
|  | **Satisfaction with breast** | **Satisfaction with BRR or abdomen** | **Satisfaction with overall outcome** | **Physical well-being** | **Psychosocial well-being** | **Sexual well-being** |  |
| **Cooke et al.^60^,**^†^  Comparative (No RT was used as the reference category)  Adjuvant RT (n=64) vs No RT (n=61)  Median difference in QOL domain scores from pre-op baseline to 1 yr post-op  Other MROC questionnaire subunits (NPRS, PROMIS-29, MPQ, GAD-7, PHQ-9) all NS | -5 NS^§^ | 0 NS | -7 NS | -4 NS^§^ | -7 NS^§^ | -4 NS^§^ | **Breast symptoms 8*** (P < 0.0001, CI 5-12)  Greater breast symptoms after RT at 1 yr post-op  Arm symptoms 15 NS^§^  Future perspective 0 NS^§^  Functional body image -3 NS^§^  Systemic therapy side effects 0 NS^§^  Sexual functioning 0 NS^§^ |
| **O’Connell et al.^58^,**^‡^  Comparative  Adjuvant RT (n=27) vs No RT (n=79)  Median QOL domain score of each cohort (exact time points of questionnaire completion not provided). Scores out of 100 (higher score indicated greater satisfaction) | **64 vs 75*** (P=0.008)  (IQR 53-73 vs 65-85)  Greater satisfaction with No RT | NA | NA | NA | NA | NA | NA |
| **Billig et al.^62^,**^†^  Longitudinal  Non-comparative  Adjuvant RT only (n=108)  Mean within-patient change in QOL domain scores from pre-op baseline to 1 and 2 yrs post-op | **1 yr: 5.8*** (P=0.018),  **2 yr: 6.0*** (P=0.047)  Greater satisfaction with breast at 1 and 2 yrs post-op | NA | NA | Chest & upper body:  1 yr: -3.0 NS,  2 yr: -2.4 NS  Abdomen:  **1 yr: -10.9*** (P < 0.001),  **2 yr: -10.1*** (P < 0.001)  Lower physical well-being (abdomen) at 1 and 2 yrs post-op | **1 yr: 4.2*** (P=0.033),  2 yr: 3.7 NS  Higher psychosocial well-being at 1 yr post-op (but NS at 2 yrs) | 1 yr: 0.2 NS,  2 yr: 0.0 NS | NA |

^†^Prospective studies.

^‡^Retrospective studies.

***Statistically significant (P < 0.05)**.

^§^Multivariate analysis.

QOL, Quality of Life; BRR, Breast Reconstruction; RT, Radiotherapy; EORTC, European Organisation for Research and Treatment of Cancer Breast Cancer-Specific Quality of Life Questionnaire (QLQ-BR23); vs, Versus; MROC, Mastectomy Reconstruction Outcomes Consortium; NPRS, Numerical Pain Rating Scale; PROMIS-29, Patient-Report Outcomes Measurement Information System Profile 29; MPQ, McGill Pain Questionnaire; GAD-7, Generalized Anxiety Disorder Scale; PHQ-9, Patient Health Questionnaire; NS; Not Significant; NA, Not Applicable/available; pre-op, Pre-operative; post-op, Post-operative; yr(s), Year(s); IQR, Interquartile Range; CI, Confidence interval.

**Table S3** Study evaluating patient-reported quality of life in delayed autologous breast reconstruction comparing neoadjuvant radiotherapy with no radiotherapy, and non-comparative study (neoadjuvant radiotherapy only)

| **Study,**  **Scoring description** | **BREAST-Q for Breast Reconstruction (BRR)** | | | | |
| --- | --- | --- | --- | --- | --- |
|  | **Satisfaction with breast** | **Physical well-being (chest & upper body)** | **Physical well-being (abdomen)** | **Psychosocial well-being** | **Sexual well-being** |
| **O’Connell et al.^58^,**^‡^  Comparative  Neoadjuvant RT (n=38) vs No RT (n=79)  Median QOL domain score of each cohort (exact time points of questionnaire completion not provided). Scores out of 100 (higher score indicated greater satisfaction) | 81 vs 75  (IQR 69-91 vs 65-85) | NA | NA | NA | NA |
| **Billig et al.^62^,**^†^  Non-comparative  Neoadjuvant RT only (n=67)  Mean within-patient change in QOL domain scores from pre-op baseline to 1 and 2 yrs post-op | **1 yr: 30.1***  (P < 0.001),  **2 yr: 32.2***  (P < 0.001)  Greater satisfaction with breast at 1 and 2 yrs post-op | 1 yr: 1.9 NS,  **2 yr: 5.8*** (P=0.007)  Higher physical well-being (chest & upper body) at 2 yrs post-op (but NS at 1 yr) | **1 yr: -9.2*** (P=0.001),  2 yr: -2.0 NS  Lower physical well-being (abdomen) at 1 yr post-op (but NS at 2 yrs) | **1 yr: 21.2***  (P < 0.001),  **2 yr: 24.1***  (P < 0.001)  Higher psychosocial well-being at 1 and 2 yrs post-op | **1 yr: 24.2***  (P < 0.001),  **2 yr: 22.8***  (P < 0.001)  Higher sexual well-being at 1 and 2 yrs post-op |

^†^Prospective studies.

^‡^Retrospective studies.

***Statistically significant (P < 0.05)**.

QOL, Quality of Life; BRR, Breast Reconstruction; RT, Radiotherapy; vs, Versus; NA, Not Applicable/available; NS, Not Significant; pre-op, Pre-operative; post-op, Post-operative; yr(s), Year(s); IQR, Interquartile Range.

**Fig. S1** Forest plot comparisons for adjuvant radiotherapy *versus* no adjuvant radiotherapy

**a** Infection; **b** wound complications (delayed wound healing/dehiscence).

**Fig. S2** Forest plot comparisons for neoadjuvant radiotherapy *versus* no neoadjuvant radiotherapy

**a** Partial flap loss; **b** total flap loss.

**Fig. S3** Forest plot comparisons for combined adjuvant and neoadjuvant radiotherapy *versus* no radiotherapy

**a** Clavien–Dindo classification (CDC) grade II complications; **b** emergency reoperation for complications; **c** partial flap loss; **d** total flap loss; **e** infection; **f** wound complications (delayed wound healing/dehiscence).
