# Supplementary figures and images for: Immediate and delayed autologous abdominal microvascular flap breast reconstruction in patients receiving adjuvant, neoadjuvant or no radiotherapy: a meta‐analysis of clinical and quality‐of‐life outcomes
Source: BJS Open. 2019 Dec 29;4(2):182–96. doi: 10.1002/bjs5.50245 (PMC7093792; doi:10.1002/bjs5.50245)

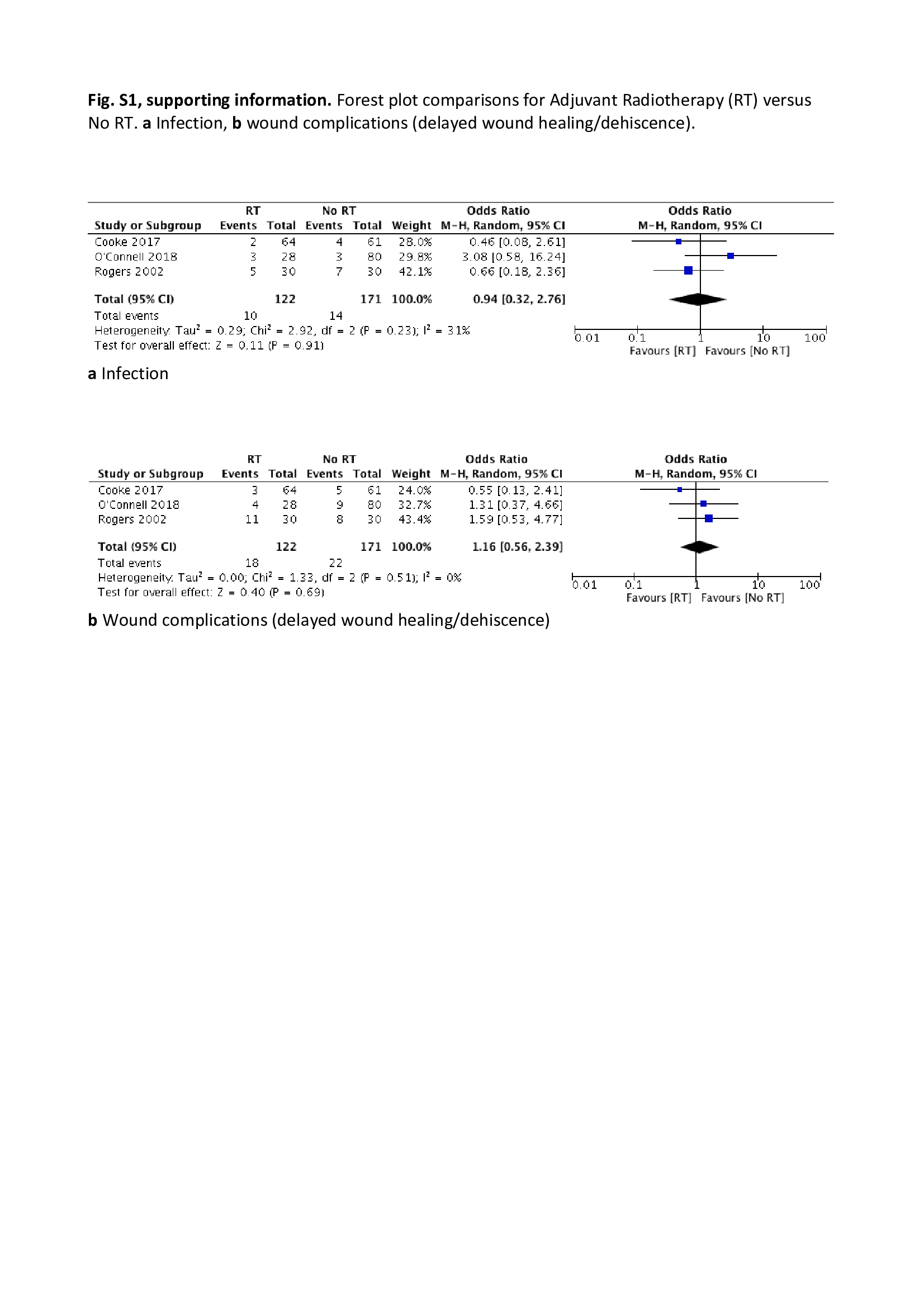

Supplement: Supplementary file 2 — Fig. S1 Forest plot comparisons for adjuvant radiotherapy versus no adjuvant radiotherapy [file BJS5-4-182-s002.tiff]

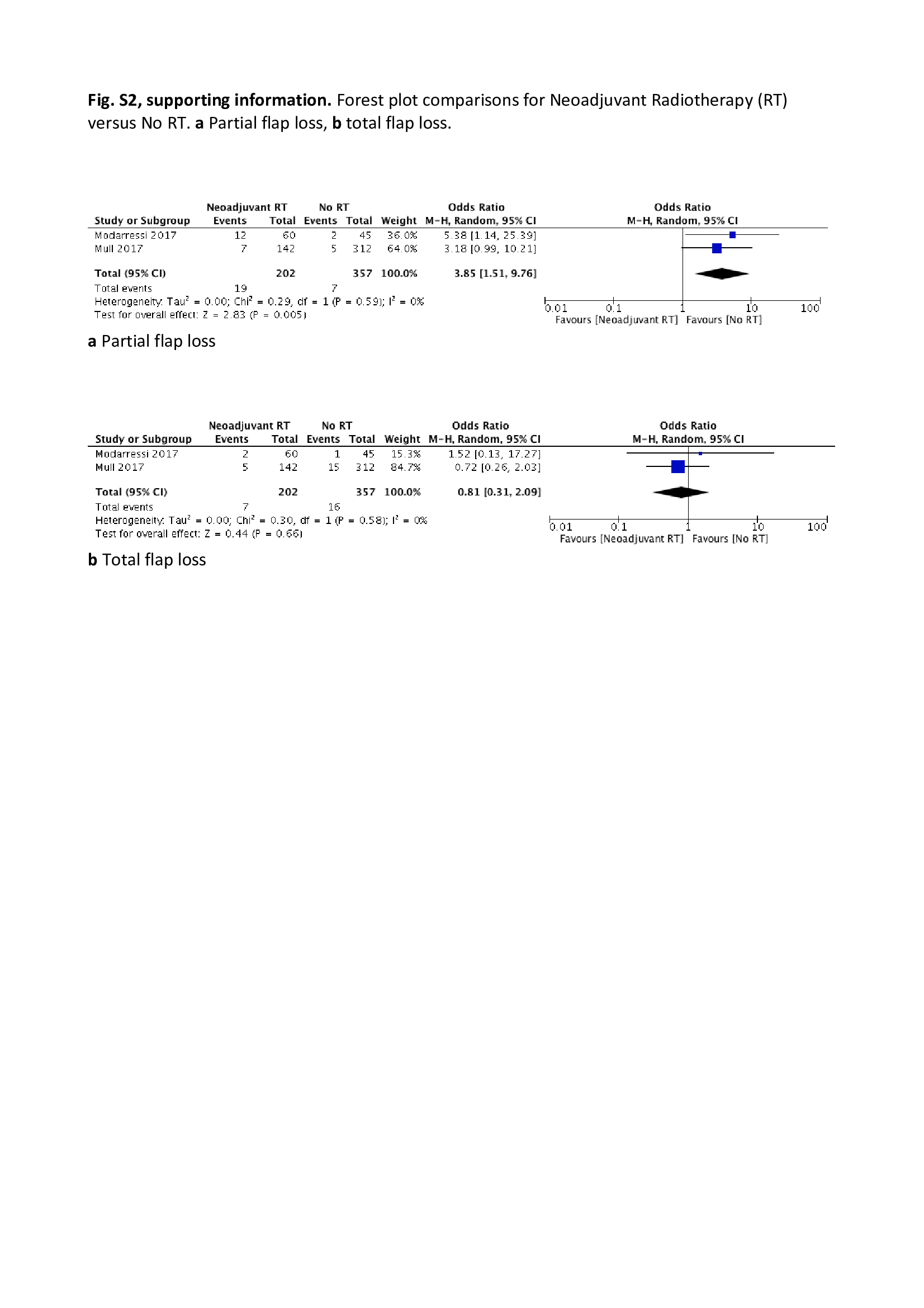

Supplement: Supplementary file 3 — Fig. S2 Forest plot comparisons for neoadjuvant radiotherapy versus no neoadjuvant radiotherapy [file BJS5-4-182-s003.tiff]

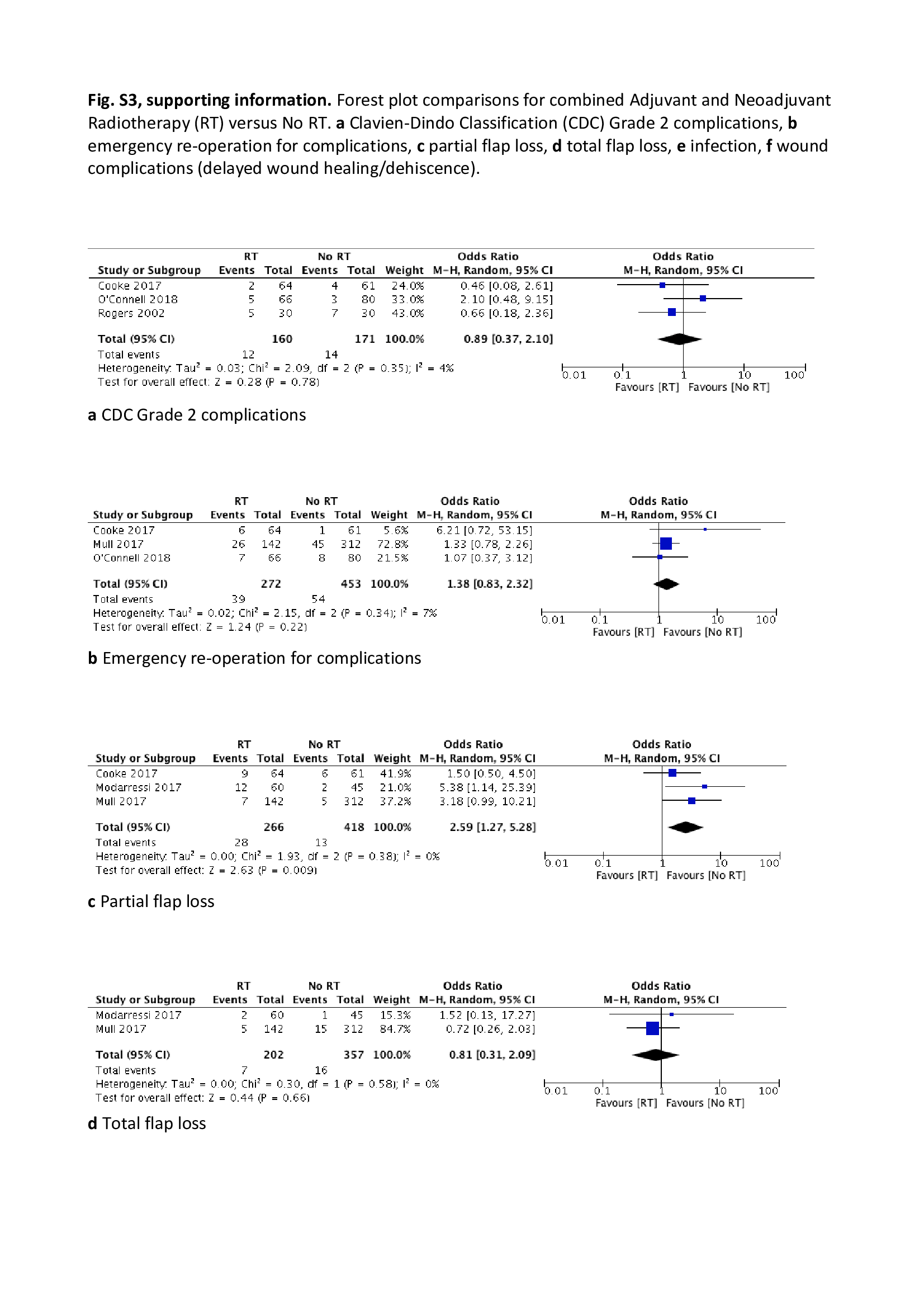

Supplement: Supplementary file 4 — Fig. S3 Forest plot comparisons for combined adjuvant and neoadjuvant radiotherapy versus no radiotherapy [file BJS5-4-182-s004.tiff]
